# Exploration on Electronic Properties of Self-Assembled Indium Nitrogen Nanosheets and Nanowires by a Density Functional Method

**DOI:** 10.3390/molecules28217358

**Published:** 2023-10-31

**Authors:** Running Zhao, Rui Chen, Hua Zhao, Fan Lin, Ju-Guang Han

**Affiliations:** 1 School of Arts and Sciences, Shanghai Dianji University, Shanghai 201306, China; 2 National Synchrotron Radiation Laboratory, University of Science and Technology of China, Hefei 230029, China

**Keywords:** electronic properties, self-assembled (InN)_12n_ materials, growth pattern, charge transfers, nano-film

## Abstract

Equilibrium geometries and properties of self-assembled (InN)_12n_ (n = 1–9) nanoclusters (nanowires and nanosheets) are studied using the GGA-PBE (general gradient approximation with Perdew–Burke–Ernzerh) method. The relative stabilities and growth patterns of semiconductor (InN)_12n_ nanoclusters are investigated. The odd-numbered nano-size (InN)_12n_ (n is odd) have weaker stabilities compared with the neighboring even-numbered (InN)_12n_ (n is even) ones. The most stable (InN)_48_ nanosheet is selected as a building unit for self-assembled nano-size film materials. In particular, the energy gaps of InN nanoclusters show an even–odd oscillation and reflect that (InN)_12n_ (n = 1–9) nanoclusters are good optoelectronic materials and nanodevices due to their energy gaps in the visible region. Interestingly, the calculated energy gaps for (InN)_12n_ nanowires varies slightly compared with that of individual (InN)_12_ units. Additionally, the predicted natural atomic populations of In atoms in (InN)_12n_ nanoclusters show that the stabilities of (InN)_12n_ nanoclusters is enhanced through the ionic bonding and covalent bonding of (InN)_12n_ (n = 1–9) nanoclusters.

## 1. Introduction

In order to develop future novel nanomaterials, the semiconducting nanostructures of materials play key roles because they uncover unique behaviors and possess widespread potential applications in energy materials and electronic and optoelectronic nanodevices [1,2,3,4,5,6,7,8,9,10,11,12,13,14,15,16,17,18]. In particular, self-assembled nanowires, which can address fundamental issues related to dimensionality, have widespread applications in optoelectronic and nano-electronic devices. The interesting properties of size-specific self-assembled nanosheets or cluster-assembled nanowires [6,8,12] allow new nanomaterials and nanodevices to be generated that can be applied to different nanoscience fields [1,2,3,6,8,10,11,12,13,14,15,16,17,18,19,20,21]. The individual properties of the magic stable cluster being used as a building unit for these self-assembled nanosheets, nanowires, or nanomembranes are actually maintained. It should be pointed out that cluster self-assembly is a spontaneous formation of well-defined and ordered structural forms; thus, reliable self-assembly techniques are surely suitable for different purposes for developing nanomaterials. However, these self-assembled nanostructures hold unique electronic properties and may acquire different properties or functions that differ from those observed for macroscale counterpart materials. The specific geometrical forms and electronic properties of self-assembled nanostructures have very important potential applications in energy nanomaterials and electronic nanomaterials [1,10,11,12,13,14,15,16,17,18,19,20,21]. Thus, semiconductor structured nanomaterials hold the potential to promote advancements in the rapidly growing technological fields.

It has been found that metal oxides (ZnO, Al_2_O_3_, and MgO), metal sulfides (Cu_3_S_3_, CdS, and ZnS), and transition metal nitrides (GaN, AlN, and InN), together with their cluster-assembled nanomaterials, are of very high interest and have been widely investigated [1,2,3,4,5,7,8,9,10,11,12,13,14,15,16,17,19,20,21], and the possible geometries and energy gaps of inner hollow semiconductor (GaN)_2n_ nanocages have been confirmed [21]. It has been found that the stable GaN and InN nanocages are good semiconductor materials with stronger electron mobility, which is consistent with experimental values [9,11,13,14]. Moreover, the relative stabilities of nano-size (InN)_2n_ and (CdS)_2n_ (n = 5–27) clusters have been studied, and the photon-to-current conversion efficiency related to the energy conversion of inner hollowed nano-size Cd_2n_S_2n_ (n = 5–27) clusters has been discussed [7,9]. Among these structured nanomaterials, ultrathin two-dimensional (2D) nanomaterials and quantum size effects have received attention because of their applications and new properties [12]. (GaN)_n_, (AlN)_n_ (n = 4–6), and (InN)_n_ clusters, together with bulk and 2D InN crystals, have been predicted theoretically [1,8,12]. The density functional theory (DFT) method confirms that AlP material, with high photoelectron absorption, is an indirect semiconductor material, whereas GaP, GaN, and AlN are direct semiconductor nanomaterials with energy band gaps predicted to be 4.51, 2.36, 1.84, and 2.01 eV, respectively. Moreover, AlP/GaP/AlN/GaN materials demonstrate potential applications [21]. Single-crystalline AlN nanotubes and nanowires, with diameter regions from 2.2 to 0.7 nm, have been explored theoretically [12]. Consequently, significant progress has been achieved in structure determination and electronic property evacuation for nanosheets and nanowires, particularly for self-assembled (InN)_12n_ superclusters or nanomembranes. Stable (InN)_12n_ units can self-assemble 2D atomic mono-layer nanosheets, nanowires, and In_12_N_12_ aggregated superclusters, with important applications for microelectronic and optoelectronic nanodevices. Interestingly, InN nanosheets, with the highest electron mobility and the lowest effective electron mass, are suitable for high-frequency and high-power nanodevices [14].

The computational simulations and experimental measurements of properties for self-assembled (InN)_12n_ superclusters originating from the (InN)_12_ units have not been fully studied due to the current experimental challenges in identifying their geometries. In order to obtain all of the possible properties and growth patterns of self-assembled (InN)_12n_ nanosheets or nanowires, to clarify the relationship between electronic structures and geometrical configurations, and to explore their important applications, a number of self-assembled (InN)_12n_ nanosheets and nanowires originating from (InN)_12_ units were designed and predetermined with DFT methods. However, geometries and electronic structures of self-assembled (In_12_N_12_)_n_ nanoclusters linked by six-membered rings had not been carried out previously. Consequently, this computational investigation focuses on the predictions and determinations of the favorable growth patterns, stabilities, geometries, and electronic properties, as well as the driving forces, of self-assembled semiconductor (InN)_12n_ nanosheets and nanowires formed by individual (InN)_12_ units bonded together by six-membered rings using established density functional methods.

## 2. Computational Details

Geometrical and electronic properties of self-assembled (InN)_12n_ nanoclusters (nanowires and nanosheets) with elemental (InN)_12_ units were combined by means of a six-membered ring, performed by DFT (density functional theory) simulations with the gradient-corrected PBE (Perdew–Burke–Ernzerh) [22] of the exchange–correlation functional within the Kohn–Sham framework of the density functional theory, as implemented in the GAUSSIAN09 RevD.01 package [23]. The exchange and the correlation part in PBE1PBE were specified as follows: The “1” stands for “1 parameter hybrid”, i.e., the functional consists of 25% from Hartree–Fock and 75% from PBE for the exchange part, and then the correlation maintains the PBE part (it is the same as PBE0 [24]). This means that the hybrid PBE0 functional compensates for part of the inaccuracy of the PBE XC by adding a fraction of the exact exchange, allowing for more accurate results in the evaluation of the energy gap. Energy gaps are calculated with increased accuracy with this method compared to “standard” PBE. The accurate electronic properties and geometries of self-assembled semiconductor (InN)_12n_ superclusters can be determined by adopting 6-31G(d) and LanL2DZ basis sets for nitrogen and indium atoms, respectively. Moreover, the PBE method is suitable for calculating the metal complexes and provides a reasonable explanation of the geometries and electronic properties.

The PBE1PBE exchange–correlation functional with 6-31G(d) and LanL2DZ basis sets for N and In atoms, respectively, is already conformed to best describing the electronic structures of InN nanomaterials [9]. Using this reliable theoretical method, the electronic and geometrical properties of inner hollowed nanoscale (InN)_2n_ (n= 6–27, 45, 54) clusters can be successfully determined [9,25]. One can surely ascertain that this method is suitable for investigating self-assembled (InN)_12n_ superclusters (or nanoclusters) and provides reliable geometrical forms and reasonable electronic properties. The optimized geometries of all self-assembled (InN)_12n_ nanoclusters were systematically determined. In addition, zero-point vibrational energy was taken into account, and the total energy of (InN)_12n_ was calculated. In order to test the stabilities of (InN)_12n_ nanoclusters (nanosheets and nanowires), their harmonic vibrational frequencies were considered and evaluated to determine the clusters’ stabilities. Zero-point vibrational energy (ZPVE) was included in the total energy and all calculations were carried out at 298.15 K under a 1 atmosphere. In addition, it is well known that the HOMO-LUMO gaps of nanoclusters can be influenced by their size; it is difficult to predict the quantum size effect by using the PBE1PBE method accurately. However, range-separated functionals can yield slight corrections of the calculated HOMO-LUMO gaps of (InN)_12n_ nanoclusters [26,27]. In this article, the simulations of HOMO, LUMO, and HOMO-LUMO gaps at the PBE1PBE level considering range-separated functionals is ignored due to the extensive computational time.

## 3. Results and Discussion

Recent experimental measurements exhibited that InN nanosheets or nanowires with the highest electron mobility and the lowest effective electron mass are widely suitable for high-power and high-frequency devices and have significant applications in optoelectronic nanodevices [1,8,28]. Consequently, studies of newly self-assembled (InN)_12n_ nanosheets and nanowires are opening interesting research fields for developing next-generation nanomaterials with unique properties. In order to self-assemble (InN)_12n_ nanoclusters with the aid of DFT methods, the selected linking or bonding atoms connecting individual (InN)_12_ units are very critical for forming specific nanomaterials with unique electronic properties. That is to say, the selected linking ligands or bonds between two individual (InN)_12_ units can lead to different geometrical structures of self-assembled (InN)_12n_ nanowires and nanosheets, resulting in different properties and applications. In order to form more stable self-assembled (InN)_12n_ nanowires and nanosheets connected by six-membered rings of individual (InN)_12_ units, in this study, the selected linking atoms in six-membered rings of an (InN)_12_ cage differed from the atom types in other six-membered rings of (InN)_12_ units. As far as the self-assembled (InN)_12n_ superclusters connected by six-membered rings are concerned, detailed studies of the self-assembled (InN)_12n_ nanosheets and nanowires were taken into account by using the PBE1PBE method.

### 3.1. Geometries and Stabilities

**Figure 1:**
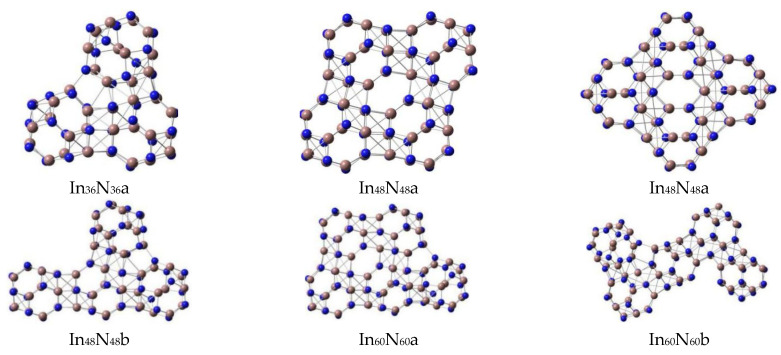

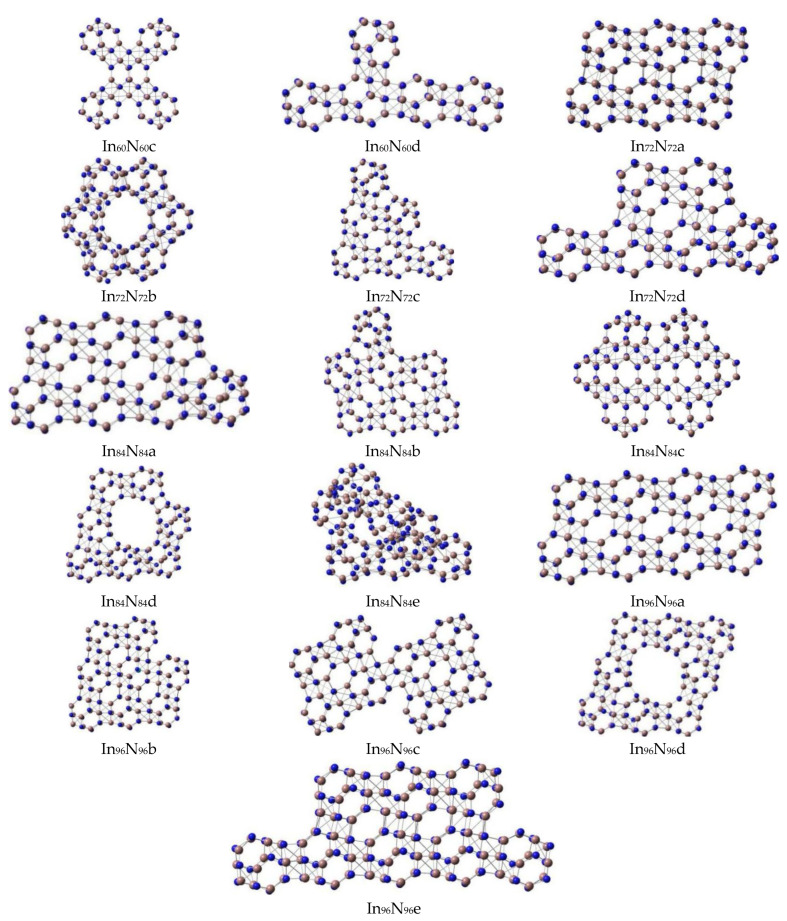
The stable (InN)_12n_ (n = 1–8) nanosheets with In_12_N_12_ units combined by their six-membered rings.

**Figure 2:**
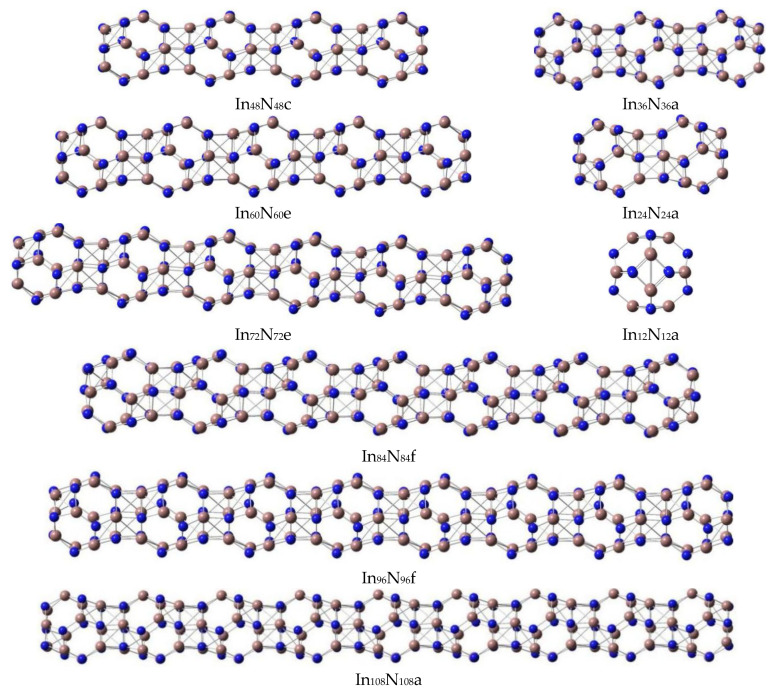
The stable (In_12_N_12_)_n_ (n = 1–9) nanowires connected by six-membered rings.

In order to provide an accurate understanding of the geometrical and electronic properties of (InN)_12n_ nanosheets with n = 1–8 (Figure 1 and Table 1) and (InN)_12n_ nanowires with n = 1–9 (Table 2 and Figure 2), beginning with the predictions of the accurate geometrical forms of (InN)_12n_ (n = 1–9) nanoclusters is required. Thus, the stable In_12_N_12_ unit was selected as an ideal building block [9] for designing various possible initial geometries of self-assembled (InN)_12n_ nanowires and nanosheets.

All possible geometries of (InN)_12n_ (n = 1–9) nanowires and (InN)_12n_ (n = 1–8) nanosheets were optimized at the PBE1PBE level with a size in the regions of 5.1~63.4 Å. The (InN)_12n_ (n = 1–9) nanoclusters (nanosheets and nanowires) were composed of many stable individual (InN)_12_ units (Figure 1 and Figure 2) connected by hexagonal prisms formed by six-membered rings in different (InN)_12_ units. In particular, the stable geometries of the (InN)_12n_ (n = 1–9) nanowires formed by self-assembly of a number of (InN)_12_ units (Figure 2) were optimized to be stable linear forms with zero dipole moments and higher point group symmetry (Table 2).

According to the calculated dipole moment of the In_24_N_24_ unit in Table 1, the optimized geometry of the In_24_N_24_ nanowire is presented as a linear structure, which was composed of two (InN)_12_ units. The N-In bond lengths in each (InN)_12_ unit of In_24_N_24_ nanowire were predicted to be 2.27 (four-membered ring) and 2.12 Å (six-membered ring). The In-N connecting distance between two (InN)_12_ units was 2.14 Å, and these values demonstrate that the stable geometry of each (InN)_12_ unit in the In_24_N_24_ nanowire was slightly changed under the interactions between two (InN)_12_ units after both (InN)_12_ units were combined. In particular, the stable (InN)_12_ geometry near the connecting part deviated slightly from its original individual form. However, the (InN)_12_ geometry, which was not involved in the connecting section, being 2.01 (or 2.00) Å for hexagons (six-member rings) and 2.09 Å for squares (four-member rings), maintained the geometrical form of its individual isolated (InN)_12_ unit. As far as the In_36_N_36_ isomers are concerned, two possible In_36_N_36_ isomers were considered and optimized as stable structures. The energetically favorable In_36_N_36_ geometry with a zero dipole moment was the linear form, which was lower in total energy than the triangle form and slightly deformed the higher point-group symmetry, reflecting that linear In_36_N_36_ geometry was the most stable one. The linear geometry of In_36_N_36_a is shown in Figure 2, and its total energy was higher than that of In_36_N_36_b, which is shown in Figure 1.

A variety of possible initial geometrical forms were optimized for In_48_N_48_ geometries. The four stable In_48_N_48_ geometries are illustrated in Figure 1 and Figure 2. For the optimized In_48_N_48_ geometries, the most stable rhombus In_48_N_48_a geometry was selected as the ground state and its dipole moment was calculated to be 0.01 Debye. The In_48_N_48_b was seen to be an (InN)_12_ cage capped on the linear In_36_N_36_ geometry. This geometry was slightly bigger in total energy than the linear In_48_N_48_c cluster. After one (InN)_12_ unit was capped on the In_48_N_48_a rhombus geometry, the newly formed nonlinear In_60_N_60_a nanosheet was generated, which was the lowest-energy structure. The In_60_N_60_b, In_60_N_60_c, and In_60_N_60_d isomers were yielded by inserting one In_12_N_12_ unit into the In_48_N_48_b structure. Moreover, the total energies of these geometries were higher than those of the linear In_60_N_60_e isomer, reflecting that the linear In_60_N_60e_ structure was more stable than those of the In_60_N_60_b, In_60_N_60_c, and In_60_N_60_d isomers; however, it was less stable than that of the In_60_N_60_a nanosheet.

According to the optimized stable In_72_N_72_ geometries, the newly formed rhombus In_72_N_72_a geometry was the most stable geometry, and its dipole moment was predicted to be 0.01 Debye, which was generated from the In_60_N_60_a nanosheets via the addition of a new In_12_N_12_ unit; moreover, this geometry deviated slightly to the higher point-group symmetry. It should be mentioned that the linear In_72_N_72_e geometry was less stable energetically than the In_72_N_72_b, In_72_N_72_c, In_72_N_72_d, and In_72_N_72_e geometries.

Following these procedures, In_84_N_84_a and In_84_N_84_b isomers, which were generated from the In_72_N_72_a geometry, were investigated. As seen in Table 1 and Table 2, it is exhibited that the calculated total energy difference between In_84_N_84_a and In_84_N_84_b was 0.092 eV, and the total energy of the In_84_N_84_b geometry was slightly higher than that of In_84_N_84_a. Consequently, In_84_N_84_a was more stable than the In_84_N_84_b nanosheet, and the In_84_N_84_a nanosheet was confirmed to be the most stable geometry. In particular, the In_84_N_84_c nanosheet was obtained by combining two In_48_N_48_a units together, and it was less stable than the In_84_N_84_b nanosheet. The In_84_N_84_e nanosheet, which deformed the plane-like form, was only higher in total energy than the linear In_84_N_84_f geometry. However, the energy gap of the linear In_84_N_84_f geometry (2.60 eV) was bigger than that (2.45 eV) of the most stable In_84_N_84_a structure. Eventually, the distinct characteristic of the energy gap of linear geometry was increased with the size of the cluster being extended. In particular, an energy gap of an In_84_N_84_f nanowire being 2.60 eV is in the range suitable for optoelectronic devices that emit in the visible region. The formation of semiconductor nanowires with an energy gap in the visible region of 1.62~3.11 eV has potential applications in the fields of nanophotonics, nanoelectronics, and medicine, which are consistent with the experimental results.

All possible In_96_N_96_ isomers were considered, and six possible In_96_N_96_ nanoclusters were optimized to be stable geometries. The stable In_96_N_96_a nanosheet was generated by capping one In_12_N_12_ unit on the In_84_N_84_a or In_84_N_84_b isomers. The In_96_N_96_a nanosheet with a dipole moment of 0.04 Debye was lower in total energy by 1.104 eV than the In_96_N_96_b nanosheet. Consequently, the In_96_N_96_a nanosheet was more stable than the In_96_N_96_b nanosheet; the formation of the In_96_N_96_c nanosheet was based on the combination of two In_48_N_48_ units, and the In_96_N_96_c nanosheet was less stable than both In_84_N_84_a and the In_84_N_84_b isomers. The interesting rhombus In_96_N_96_d nanosheet yielded a big hole in the center; this stable isomer can be suitable for hosting new clusters or molecules for application in porous nanomaterials. According to the calculated results in Table 1 and Figure 1, the In_96_N_96_e nanosheet was also obtained from the In_84_N_84_a nanosheet, and this structure was more stable than both the In_96_N_96_c and the In_96_N_96_d nanosheets. Finally, the linear In_96_N_96_ geometry is mentioned, and this isomer had the same energy gap (2.60 eV) as the linear In_72_N_72_ and In_84_N_84_ isomers (Figure 2 and Table 2). In particular, the energy gap of the In_108_N_108_ nanowire was determined to be 2.59 eV, which was slightly smaller than those of the In_72_N_72_, In_84_N_84_, and In_96_N_96_ nanowires.

### 3.2. Relative Stabilities

The relative stabilities of (InN)_12n_ nanoclusters consisting of different In and N atoms were investigated based on the average InN diatomic binding energies (E_b_(n)) as well as the calculated fragmentation energies (D(n, n−1)), which are defined as

(1) $$E_{b}(n)=\frac{12n*E_{T}(InN)-E_{T}[(InN{)}_{12n}]}{12n}$$

(2) $$D(n,n-1)=E_{T}[{\left(InN\right)}_{12(n-1)}]+E_{T}{\left(InN\right)}_{12}-E_{T}[{\left(InN\right)}_{12n}]$$

where E_T_[(InN)_12n_], E_T_(InN), and E_T_[(InN)_12(n−1)_] are the calculated total energies of the respective (InN)_12n_ and (InN)_12(n−1)_ nanoclusters, together with the InN dimer, with zero-point vibration energies being taken into accounts. The total energy of the InN diatom was predicted to be −56.424257 Hartree.

**Figure 3:**
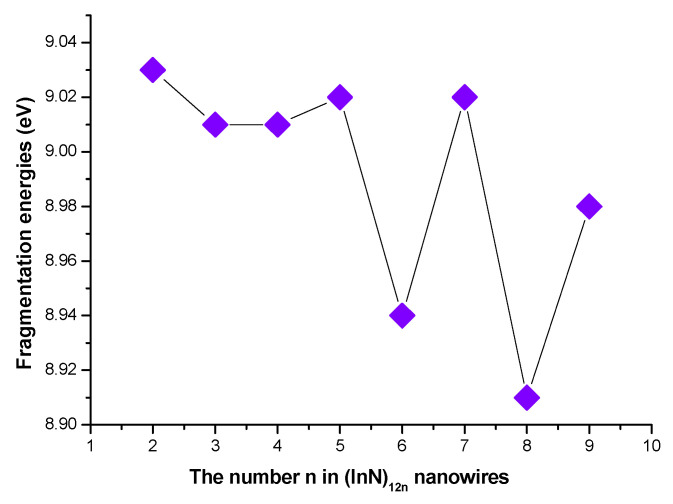
The calculated fragmentation energies of (InN)_12n_ nanowires.

Although the (InN)_12n_ nanowires were relative weak in stability compared to the (InN)_12n_ nanosheets, the promising potential applicability of (InN)_12n_ nanowires is very unique because a newly type of semiconductor (InN)_12n_ nanowire in a radius within the region of a few tens of nanometers is considered to be the ideal candidate for further developing optoelectronic and electronic nanodevices. Thus, the relative stabilities of (InN)_12n_ nanowires are considered and discussed in detail in this study due to their unique semiconductor properties. According to our calculated relative stabilities of (InN)_12n_ nanowires connected by six-membered rings, the average InN diatomic binding energy (E_b_(n)) of (InN)_12n_ nanowires increased continually to the maximum value of 4.714 eV when the size of (InN)_12n_ nanowires varied from n = 1 to n = 9. In order to demonstrate the reliability of the averaged InN diatomic binding energies of (InN)_12n_ nanowires, the fragmentation energies (D(n,n−1)) of (InN)_12n_ nanowires were also taken into accounts. As seen from the curves of the fragmentation energies as a function of size (Figure 3 for (InN)_12n_ nanowires), the apparent tendency for (InN)_12n_ nanowires was that their fragmentation energies decreased as the size of (InN)_12n_ nanowires varied from n = 1 to n = 9 (Figure 3). In general, the calculated values of the fragmentation energies for the (InN)_12n_ nanowires exhibited an alternating even–odd oscillation, with the even-numbered (InN)_12n_ nanowire (*n* is even) being smaller in fragmentation energy than its odd-numbered neighboring (InN)_12n_ nanowires (n is odd), reflecting that the odd-numbered semiconductor (InN)_12n_ nanowires had stronger stability than their neighboring even-numbered semiconductor (InN)_12n_ nanowires.

**Figure 4:**
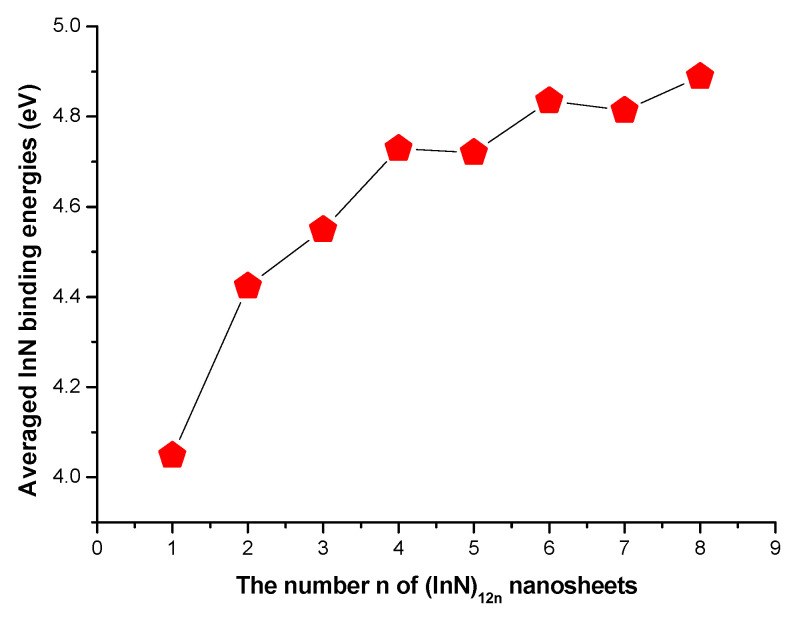
The calculated average InN diatomic binding energies of (InN)_12n_ nanosheets.

The average InN diatomic binding energies of the stable (InN)_12n_ nanoclusters (including nanowires and nanosheets) are listed in Table 1 and Table 2 and shown in Figure 4. Based on the calculated average InN diatomic binding energy for (InN)_12n_ nanoclusters, the magic numbers of stabilities for (InN)_12n_ nanoclusters were assigned at n = 2, 4, 6, and 8, reflecting that the even-membered semiconductor (InN)_12n_ nanoclusters (n is even) were apparently more stable compared with their neighboring odd-numbered n in (InN)_12n_ (n > 2) units.

**Figure 5:**
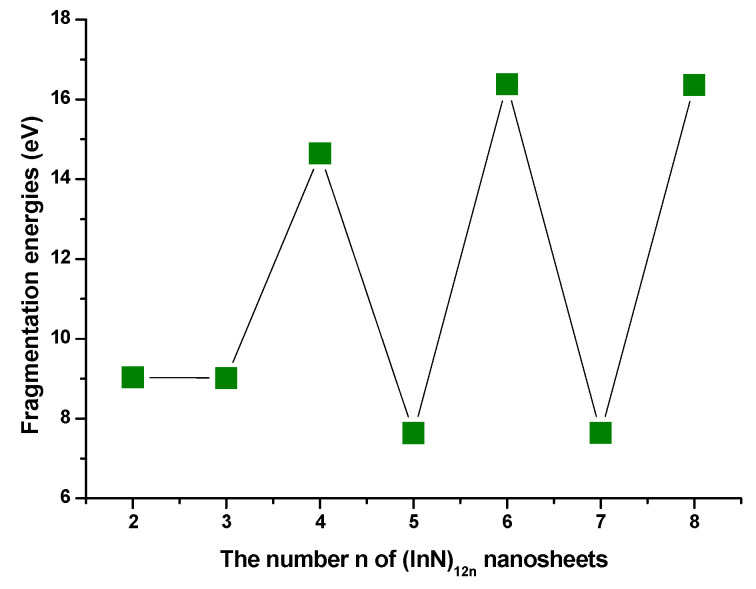
The calculated fragmentation energies of (InN)_12n_ nanosheets.

Based on the calculated D(n,n−1) of (InN)_12n_ nanoclusters (nanowires and nanosheets) shown in the curve in Figure 5, the calculated fragmentation energies for (InN)_12n_ nanoclusters had the same tendency as those of (InN)_12n_ nanowires mentioned above, with the calculated values of fragmentation energies for (InN)_12n_ nanoclusters generally showing an appealing even–odd oscillation. On the contrary, the even-numbered semiconductor (InN)_12n_ nanoclusters (where n is even) had higher fragmentation energy than the odd-numbered ones, suggesting that the even-numbered nano-size (InN)_12n_ (where n is even) clusters had stronger stability compared with their odd-numbered (InN)_12n_ neighbors. The remarkable peaks were n = 4, 6, and 8. Interestingly, the In_72_N_72_ and In_96_N_96_ nanosheets, with n = 6 and 8, respectively, were composed of individual (InN)_48_ (n = 4) units, indicating that In_72_N_72_ and In_96_N_96_ nanosheets were generated by the primarily newly formed In_48_N_48_ nanosheets; thus, one can infer that even-numbered (InN)_12n_ can be detected more easily with mass spectroscopy experimental measurements with remarkably large abundances than the neighboring odd-numbered (InN)_12n_ ones. More importantly, the (InN)_12n_ nanosheet with n = 4 was assigned as a newly formed self-assembled building block for new film nanosheets. Eventually, the (InN)_48_a nanosheet was selected as the newly formed building block for self-assembled (InN)_12n_ semiconductor film nanosheets (or nanomembranes), which have an important application in microelectronic and optoelectronic nanodevices. They can form the magnetic nanomembrane after magnetic metal atoms or ions are inserted in a suitable hole in (InN)_12n_ nanosheets. 

### 3.3. HOMO-LUMO Gaps

**Figure 6:**
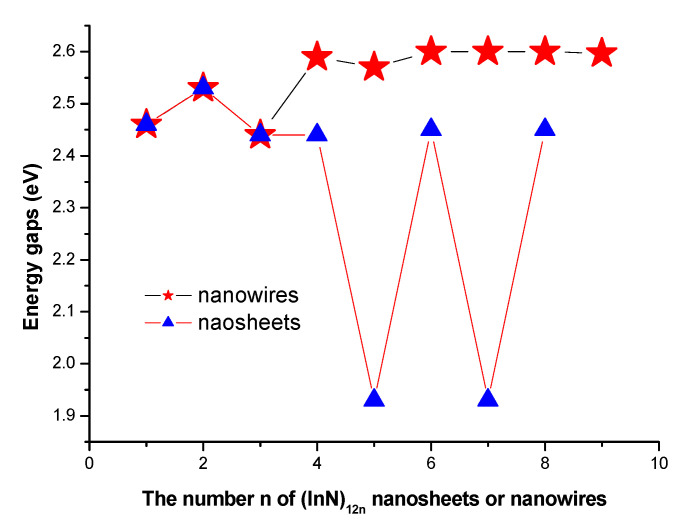
Energy gaps of (InN)_12n_ nanowires or nanosheets as a function of the size of the nanowires or nanosheets, respectively.

The electronic properties of self-assembled (InN)_12n_ nanoclusters (including both nanowires and nanosheets) associated with the calculated energy gap values between HOMO (the highest occupied molecular orbital level) and LUMO (the lowest unoccupied molecular orbital level) were investigated. The predicted HOMO-LUMO energy gaps of (InN)_12n_ nanoclusters are summarized in Table 1 and Table 2 and shown in Figure 6.

Based on a theoretical point of view, the advantages of (InN)_12n_ nanowires are that they are ideal models for further investigation and development of optoelectronic and electronic nanodevices. In order to reveal the geometrical and electronic properties of (InN)_12n_ nanowires, DFT studies were performed. According to the calculated energy gaps of (InN)_12n_ nanowires listed in Table 1 and shown in Figure 6, it is obvious that the obtained energy gap values of (InN)_12n_ nanowires were oscillatory, increasing at the small size and finally reaching a maximum of 2.60 eV at the large size region of n = 9. Obviously, the calculated energy gap values of (InN)_12n_ nanowires connected by six-membered rings varied slightly when the size of (InN)_12n_ nanowires changed from n = 1 to n = 9. This varying tendency of the energy gaps in semiconductor (InN)_12n_ (n = 1–9) nanowires is actually consistent with that of inner hollow InN nanoclusters [9]. Therefore, the (InN)_12n_ nanowires almost maintained the energy gap properties of an individual In_12_N_12_ unit; consequently, the variations in the energy gaps in (InN)_12n_ (n = 1–9) nanowires were indeed small when the size n of (InN)_12n_ nanowires varied from n = 1 to n = 9. The varying tendency of the energy gaps in (InN)_12n_ nanowires may have directly led to a quantum size effect. Moreover, the slight variations in the energy gaps for the semiconductor (InN)_12n_ (n = 1–9) nanowires revealed that the (InN)_12n_ nanowires possessed the analogue characteristics and properties of individual (InN)_12_ units [9]. The calculated energy gaps of the semiconductor (InN)_12n_ nanowires at the approved 2.44~2.60 eV regions were obviously within the visible light region, indicating that the size of the nanowires generated small influences on their energy gaps and reflecting that this kind of (InN)_12n_ nanowire can give rise to the widespread applications in the fields of nanophotonics and nanoelectronics. Moreover, the unique HOMO-LUMO properties of semiconductor (InN)_12n_ nanowires are receiving extremely noteworthy attention and promoted research, resulting in the opportunity for the widespread development of nanodevices and surface sciences.

The energy gap values for (InN)_12n_ nanoclusters (nanowires and nanoclusters) were calculated and are shown in Figure 6. According to the calculated energy gap values of (InN)_12n_ nanoclusters, it is exhibited that the obtained energy gaps of (InN)_12n_ nanosheets within the range of 1.93~2.53 eV depended on the varied size of the nanosheets, reflecting that cluster-assembled (InN)_12n_ nanosheets are good semiconductor optoelectronic or energy nanomaterials and give rise to attractive optical and electrooptical properties. The variation in the HOMO-LUMO gaps of (InN)_12n_ nanowires was smaller than that of (InN)_12n_ nanosheets as their size was extended. The HOMO-LUMO gaps (energy gaps) of even-numbered (InN)_12n_ nanosheets were bigger than those of the neighboring odd-numbered (InN)_12n_ nanosheets. The maximum value of the energy gap (2.53 eV) was assigned as n = 2, and the corresponding (InN)_24_ nanosheet had the strongest chemical stability. The minimum values of energy gaps (1.93 eV) were assigned as n = 5 and n = 7, and the (InN)_60_ and (InN)_72_ nanosheets had the weakest chemical stability and the strongest chemical activity, respectively. Moreover, the relative stabilities of (InN)_12n_ nanosheets with n = 5 and n = 7 were weakened compared to the neighboring (InN)_12n_ nanosheets in that their nanostructures could be elucidated as smaller energy gaps with stronger chemical activity, reflecting that the surfaces of the (InN)_12n_ nanosheets with n = 5 and n = 7 could react easily with a new (InN)_12_ unit, and stable (InN)_12(n+1)_ nanosheets were achieved. This process is a cluster–cluster chemical reaction. In fact, the growth and formation of new nanoclusters (nanosheets and nanowires) are deeply involved in the catalytic process; in the other words, the cluster-assembled nanoclusters (nanosheets and nanowires) on the surface of film actually undergo chemical processes. Thus, (InN)_12n_ with n = 4, 6, and 8 had remarkably larger abundances in experimental measurements due to their chemical stabilities. This finding is consistent with our discussions on relative stabilities in this manuscript. Moreover, the energy gaps of (InN)_12n_ nanosheets are more favorable for optoelectronic applications than the reported experimental and theoretical findings [14,15,16,17]. These dimensionally uniform self-assembled (InN)_12n_ nanosheets or nanowires may surmount the difficulties of lower efficiency, lower power, and lower quantum effect in monolayer (InN)_12n_ nanosheets.

### 3.4. Natural Charge Population Analysis

**Figure 7:**
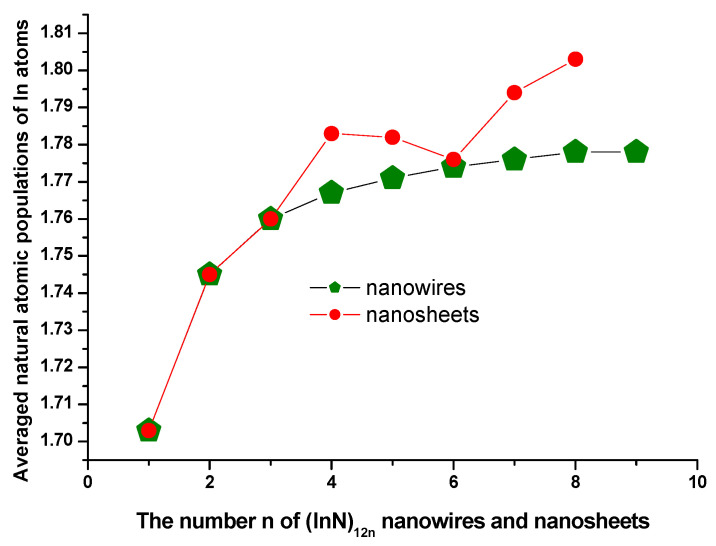
The average natural atomic populations of In atoms in (InN)_12n_ nanosheets or nanowires.

The average natural atomic charge populations of indium atoms in the (InN)_12n_ nanowires and nanosheets were obtained at the PBE1PBE level. The calculated average natural atomic charge populations for indium atoms are plotted in Figure 7. Based on the average natural atomic charge populations of indium atoms for all sizes of (InN)_12n_ nanoclusters (including nanowires and nanosheets) tabulated in Table 1 and Table 2, it is evident that the charges in the self-assembled (InN)_12n_ nanoclusters were transferred from the indium atoms to their neighboring nitrogen atoms, reflecting that the surface In atom in (InN)_12n_ nanoclusters behaved like a Lewis acid site, whereas the electronegative nitrogen atoms behaved as a Lewis base site, and that they are appropriate sites for possible carbonylation by a CO_2_ molecule. The In and N atoms in the (InN)_12n_ nanoclusters were chemically bonded with oxygen and carbon atoms, respectively, in CO_2_. Thus, (InN)_12n_ nanoclusters are suitable for decomposing a CO_2_ molecule. As can be seen from Table 1 and Table 2 and Figure 7, there was an increasing trend of the average natural atomic charge population of indium atoms in (InN)_12n_ nanoclusters with the increasing size of (InN)_12n_ nanoclusters; the electronic charges in the N atoms originated from the neighboring In atoms. As far as the average natural atomic charge populations of indium atoms in (InN)_12n_ nanowires are concerned, the maximum average natural atomic charge population values of In atoms of 1.774 and 1.778 corresponded to two remarkable peaks of (InN)_12n_ nanowires with n = 6 and n = 8, respectively, at the curve in Figure 7. However, the local maximum average natural atomic charge population values for indium atoms in (InN)_12n_ nanosheets of 1.783 and 1.803 corresponded to two remarkable peaks with n = 4 and n = 8, respectively, in Figure 7. Electrostatic interactions among all In and N atoms in (InN)_12n_ nanoclusters resulting from charge transfers increased when the size of (InN)_12n_ nanoclusters increased; therefore, the electrostatic interaction enhanced the stabilities of (InN)_12n_ nanoclusters. Furthermore, the ionic bonding formed between the In and N atoms in (InN)_12n_ nanowires or nanosheets eventually enhanced the stabilities of (InN)_12n_ nanowires or nanosheets.

## 4. Conclusions

As for the (InN)_12n_ nanoclusters consisting of numerous In_12_N_12_ units that were connected by six-membered rings, a series of (InN)_12n_ nanowires or nanosheets was taken into account and their molecular structures and properties were elucidated and investigated at the PBE1PBE level. According to the calculated average InN diatomic binding energy and fragmentation energy, the relative stabilities of (InN)_12n_ nanoclusters (nanowires and nanosheets) were studied, exhibiting that the even-numbered (InN)_12n_ nanoclusters were obviously stronger in stability than their odd-numbered (InN)_12n_ neighbors. The remarkable peaks were shown at n = 4, 6, and 8. Interestingly, the most stable In_72_N_72_ and In_96_N_96_ nanosheets consisted of (InN)_48_ nanosheets (n = 4); consequently, the (InN)_48_ nanosheet was confirmed to be a newly formed elemental building unit for cluster-assembled nanoscale film materials. With the nanowire size increasing from n = 1 to n = 9, a number of changes in the energy gaps of the (InN)_12n_ nanowires were noted. Interestingly, the HOMO-LUMO gaps of (InN)_12n_ nanowires at the region of 2.44~2.60 eV were within the visible light region. Furthermore, the slight variation in HOMO-LUMO gaps for (InN)_12n_ nanowires reflect that (InN)_12n_ nanowires maintained their property of an individual (InN)_12_ unit and reveal an important implication for the development of nanodevice prototypes. In summary, the energy gaps for (InN)_12n_ nanosheets and nanowires were calculated, and the gaps of even-numbered (InN)_12n_ nanoclusters were bigger compared with those of their odd-numbered (InN)_12n_ neighbors. In addition, the obtained averaged natural atomic populations of In atoms in (InN)_12n_ nanowires or nanosheets exhibited that charge transfers, together with ionic bonding, generally increased when the size of (InN)_12n_ nanoclusters increased, and ionic bonding in (InN)_12n_ nanoclusters existed with covalent bonding. The obtained charge transfers in (InN)_12n_ nanoclusters reflect that the In atoms of (InN)_12n_ nanoclusters behaved like a Lewis acid site, whereas the electronegative N atoms acted like a Lewis base site, and they can be appropriate sites for possible carbonylation of CO_2_ molecules.

## Figures and Tables

**Table 1:** Symmetry point groups (sym), average natural charges of In atoms (NC), dipole moment (Dip), isomers (iso), HOMO and LUMO gaps (E_gap_), average InN diatomic binding energies (E_b_(n)), fragmentation energies (D(n, n–1)), lowest frequencies (Freq), and total energies (E_T_) of the most stable (InN)_12n_ (n = 1–8) nanosheets. The stable geometries with In_12_N_12_ connected by six-membered rings.

Systems	Sym	Iso	NC	Dip	HOMO	LUMO	Freq	E_gap_	ET	Eb(n)	D(n, n−1)
				Debye	Hartree	Hartree	cm^−1^	eV	Hartree	eV	eV
In_12_N_12_	T_h_	a	1.703	0.00	−0.22082	−0.13050	71.3	2.46	−678.876339	4.048	
In_24_N_24_	C_1_	a	1.745	0.00	−0.21941	−0.12626	35.5	2.53	−1358.084415	4.424	9.03
In_36_N_36_	S_6_	a	1.760	0.01	−0.22161	−0.13177	20.3	2.44	−2037.291826	4.549	9.01
	C_1_	b	1.755	5.21	−0.20426	−0.13521	26.4		−2037.234748		
In_48_N_48_	C_1_	a	1.783	0.01	−0.21476	−0.12515	27.8	2.44	−2716.706404	4.729	14.645
	C_1_	b	1.764	5.61	−0.20392	−0.13396	17.3		−2716.439412		
In_60_N_60_	C_1_	a	1.782	4.96	−0.20447	−0.13361	18.0	1.93	−3395.863320	4.720	7.635
	C_1_	b	1.598	6.99	−0.20888	−0.13552	10.2		−3395.596897		
	C_1_	c	1.767	0.01	−0.21577	−0.12998	10.9		−3395.595081		
	C_1_	d	1.769	1.69	−0.21508	−0.12642	10.0		−3395.652494		
In_72_N_72_	C_1_	a	1.776	0.01	−0.21425	−0.12415	18.4	2.45	−4075.341547	4.835	16.377
	C_1_	b	1.776	5.02	−0.23520	−0.12284	13.0		−4074.968946		
	C_1_	c	1.781	6.22	−0.20437	−0.13344	14.3		−4075.029513		
	C_1_	d	1.783	4.05	−0.20435	−0.13351	12.9		−4075.067931		
In_84_N_84_	C_1_	a	1.794	4.88	−0.20397	−0.13304	12.5	1.93	−4754.498871	4.814	7.646
	C_1_	b	1.794	6.24	−0.20149	−0.13028	15.2		−4754.495491		
	C_1_	c	1.681	4.54	−0.20426	−0.13145	13.2		−4754.455641		
	C_1_	d	1.779	5.61	−0.20761	−0.12881	9.5		−4754.198775		
	C_1_	e	1.776	7.18	−0.20362	−0.13557	9.9		−4754.111104		
In_96_N_96_	C_1_	a	1.803	0.04	−0.21351	−0.12356	12.0	2.45	−5433.976634	4.889	16.365
	C_1_	b	1.802	1.91	−0.21697	−0.12618	11.4		−5433.936053		
	C_1_	c	1.791	0.20	−0.21141	−0.12803	6.8		−5433.655668		
	C_1_	d	1.795	0.02	−0.20682	−0.12777	10.6		−5433.515520		
	C_1_	e	1.793	1.71	−0.21340	−0.12581	9.3		−5433.712151		

**Table 2:** Symmetry point groups (sym), average natural charges of In atoms (NC), dipole moment (Dip), isomers (Iso), HOMO and LUMO gaps (E_gap_), average InN diatomic binding energies (E_b_(n)), fragmentation energies (D(n, n−1)), lowest frequencies (Freq), and total energies (E_T_) of the most stable (InN)_12n_ (n = 1–9) nanowires. The stable geometries with In_12_N_12_ connected by six-membered rings.

Systems	Sym	Iso	NC	Dip	HOMO	LUMO	Freq	E_gap_	E_T_	E_b_(n)	D(n, n−1)
				Debye	Hartree	Hartree	cm^−1^	eV	Hartree	eV	eV
In_12_N_12_	T_h_	a	1.703	0.00	−0.22082	−0.13050	71.3	2.46	−678.876339	4.048	
In_24_N_24_	C_1_	a	1.745	0.00	−0.21941	−0.12626	35.5	2.53	−1358.084415	4.424	9.03
In_36_N_36_	S_6_	a	1.760	0.01	−0.22161	−0.13177	20.3	2.44	−2037.291826	4.549	9.01
In_48_N_48_	C_1_	c	1.767	0.00	−0.21813	−0.12329	12.4	2.59	−2716.498627	4.611	9.01
In_60_N_60_	C_1_	e	1.771	0.04	−0.21783	−0.12264	8.5	2.57	−3395.706352	4.649	9.02
In_72_N_72_	C_1_	e	1.774	0.01	−0.21772	−0.12218	5.2	2.60	−4074.911066	4.673	8.94
In_84_N_84_	C_1_	f	1.776	0.02	−0.21755	−0.12193	3.9	2.60	−4754.118833	4.691	9.02
In_96_N_96_	C_1_	f	1.778	0.01	−0.21741	−0.12181	2.7	2.60	−5433.322507	4.703	8.91
In_108_N_108_	C_1_	a	1.778	0.01	−0.21724	−0.12178	1.8	2.597	−6112.528888	4.714	8.98

## Data Availability

The data available are published the Tables of this article.
